# Decreased levels of PTCSC3 promote the deterioration of prostate cancer and affect the prognostic outcome of patients through sponge miR-182-5p

**DOI:** 10.1186/s12894-024-01531-7

**Published:** 2024-07-12

**Authors:** Lin Cheng, Shuhui Li, Deqi Jiang, Rongkai Sun, Yueshan Wang, Jianchao Zhang, Qiang Wei

**Affiliations:** 1 Department of Urology Surgery, Shandong Provincial Third Hospital, No.12, Wuyingshan Middle Road, Tianqiao District, Jinan, Shandong 250031 China; 2 Department of Joint Surgery, Shandong Provincial Third Hospital, Jinan, Shandong 250031 China; 3 Department of Urology Surgery, The 960th Hospital of the PLA Joint Logistics Support Force, Jinan, Shandong 251100 China

**Keywords:** PTCSC3, miR-182-5p, Prostate cancer, Prognosis

## Abstract

**Background:**

Prostate cancer, characterized by its insidious onset and short overall survival, and has seen a rise in incidence over recent decades. This study aims to investigate the expression and molecular mechanism of lncRNA PTCSC3 (PTCSC3) in prostate cancer in order to develop new prognostic and therapeutic biomarkers.

**Methods:**

The level of PTCSC3 in serum and cell samples of prostate cancer was quantitatively measured using RT-qPCR assays. The correlation between the variation in PTCSC3 levels and clinical indicators of patients was evaluated. The survival status of the prostate cancer patients included in the study was evaluated using Kaplan-Meier curve and multivariable Cox analysis. The impact of PTCSC3 overexpression on cell growth and activity was revealed by CCK-8 and Transwell assays. The targeting relationship between PTCSC3 and miR-182-5p was determined by bioinformatics prediction and luciferase activity.

**Results:**

PTCSC3 was found to be downregulated in prostate cancer, and its low levels were associated with short overall survival in patients. It influenced the progression of prostate cancer by targeting miR-182-5p. Increasing PTCSC3 levels suppressed the proliferation, migration and invasion levels of cells, and miR-182-5p mimic counteracted PTCSC3’s effects on cells.

**Conclusions:**

As a potential prognostic biological factor for prostate cancer, PTCSC3 may regulate the progression of prostate cancer by sponging miR-182-5p and affect the prognosis of patients.

## Background

As a tumor occurring in the male genitourinary system, prostate cancer has garnered a lot of attention due to its rising occurrence and fatality rates [1]. According to 2020 data, prostate cancer ranks fourth in incidence across Europe, with approximately 470,000 cases, making up 11.75% of new diagnoses [2]. Through the statistics and analysis of cancer patients in China, the number of prostate cancer patients has dramatically increased over the past two decades, and its fatality rate is among the top five [3, 4]. With the application of detection methods like prostate cancer-specific antigen, the number of patients with early diagnosis has increased, which is beneficial to treatment outcomes [5]. However, because prostate cancer progresses slowly and early-stage symptoms are not noticeable, many patients miss the optimal treatment window, leading to poor survival rates.

To address the challenges in the treatment and prognosis of prostate cancer, molecular approaches may become the optimal choice, in addition to radical surgical resection, endocrine therapy, and chemotherapy [6, 7]. Long non-coding RNAs (lncRNAs) have been confirmed to play a role in various biological processes including tumors [8]. In prior research, the amplification or deletion of lncRNA has been found to be crucial for tumor development. For instance, PTCSC3 located on chromosome 14q.13.3 was first discovered to be abnormally low in thyroid cancer [9, 10]. PTCSC3 also reflects its regulatory role in gastric cancer, oral cancer, and cervical cancer [11–13]. However, the function of PTCSC3 in prostate cancer requires further exploration.

In this paper, we verified that the suppressor PTCSC3 is downregulated in prostate cancer. This was determined by measuring PTCSC3 in serum and cell samples. From the expression trend of PTCSC3, we explored its impact on the progression of prostate cancer and patient prognosis. This was achieved through clinical analysis, cell transfection, and in vitro assays. In addition, lncRNAs are believed to perform their sponge role by binding to microRNAs (miRNAs) [14]. To investigate this, we used bioinformatics analysis to predict and evaluate the downstream targets of PTCSC3. This allowed us to uncover the molecular mechanism of PTCSC3 in controlling prostate cancer progression. This implies that PTCSC3 may serve as a potential biomarker for prostate cancer. This opens up new possibilities for treatment and survival extension in patients.

## Methods

### Inclusion of prostate cancer patients

A total of 125 prostate cancer patients who met the requirements and 85 healthy volunteers undergoing physical examination at Shandong Provincial Third Hospital (July 2016 to July 2018) were included. Specialist pathologists diagnosed the prostate cancer patients. Patients with complications or those who had been previously treated were excluded.

This study received support and approval from the Ethics Organization of Shandong Provincial Third Hospital. All participants understood the study’s objectives and provided their written informed consent.

### Samples processing and storage

The obtained blood samples underwent high-speed centrifugation (4000 rpm, 15 min) at a 4 °C to separate tumor serum samples from normal serum samples. To ensure consistency in subsequent experiments, the serum was stored in a refrigerator at -80 °C.

### Cell selection and culture

Prostate cancer cells (CWR-R1, LNCaP, DU-145, PC-3) and control prostate epithelial cells RWPE-2 involved in the experiments were provided by ATCC (Rockville, MD). All cells were grown in DMEM medium (Invitrogen, USA) enriched with 10% fetal bovine serum (FBS) inside a 37 °C humidity incubator.

### Cell transfection

The PTCSC3 overexpression fragment (pcDNA3.1-PTCSC3), control mimic fragment (mimic NC) and miR-182-5p mimic fragment (miR-182-5p mimic) were prepared by GenePharma (Shanghai, China) and cloned into the pcDNA3.1 vector. Cells were transfected with Lipofectamine 3000 (Invitrogen, USA), and the above vectors were transfected into CWR-R1 or DU-145 cells, and the cultures were continued at a temperature of 37 °C.

### RNA isolation and RT-qPCR

RNA was isolated from serum and cell specimens by TRIZOL reagent (Thermo Fisher Scientific, USA), and its quality was then assessed with a spectrophotometer. cDNA was synthesized from RNA under the direction of a reverse transcription kit (Promega, USA). RT-qPCR assays were carried out using the SYBR^®^GREEN qPCR Super Mix Kit (Vazyme, USA), a cDNA configuration system, and the Applied Biosystems^®^7500 detection system. GAPDH and U6 were RNA internal controls for quantifying PTCSC3 and miR-182-5p. The relevant primer sequences were: PTCSC3, F: 5’-AAACTCCAGGGCTTGAAC-3’ and R: 5’-ATTACGGCTGGGTCTACCT-3’; miR-182-5p, F: 5’-CACTTTTGGCAATGGTAGAA-3’ and R: 5’- ATGGTTTTGACGACTGTGTG-3’; GAPDH, F: 5’-GCAAGAGCACAAGAGGAA-3’ and R: 5’-TGTGAGGAGGGGAGATTC-3’; U6, F: 5’-GCTTCGGCAGCACATA-3’ and R: 5’- ATGGAACGCTTCACGA-3’. Each group had three parallels, and the procedure was repeated three times.

### Cell growth assay

Cells were seeded in 96-well plates at a density was adjusted to 3 × 10^4^ cells/well and cultured at 37 °C. Then CCK-8 reagent (Sigma, USA) was added at specific time points of 0 h, 24 h, 48 h, and 72 h. After an additional 2-h incubation, the absorbance value was read at 450 nm using a microplate reader (Bio-Tek, USA) and the proliferation curve was then plotted.

### Transwell assays

The migration and invasion abilities of the cells were measured by Transwell assay. For the migration assay, cells were cultured at a density of 3 × 10^5^ cells /well in serum-free DMEM medium and placed in the upper layer. After a culture period of 24–48 h, the cells in the lower layer (DMEM medium containing FBS) were collected. Five random locations were chosen for counting under a microscope. For the invasion assay, the upper layer was coated with Matrigel (BD, USA), and the remaining steps were identical to the ones above.

### Luciferase reporter assay

The wild-type PTCSC3 (WT) was constructed by inserting the binding sites of PTCSC3 and miR-182-5p into the pmirGLO vector, and the mutation sites were cloned to generate mutant-type PTCSC3 (MUT). The PTCSC3-WT/MUT reporter vector and either mimic-NC or miR-182-5p mimic were co-transfected into CWR-R1 cells using the Lipofectamine 3000 reagent. After 48 h of transfection, luciferase activity was evaluated by dual-luciferase reporter kit (Promega, USA). Each group was set up in triplicate and the entire process was repeated three times.

### Bioinformatics analysis

The downstream targets of PTCSC3 were predicted using the lncRNASNP2, lncRNASNPv3, and LncBook2.0 datasets. The binding sites between PTCSC3 and miR-182-5p were queried through the lncRNASNPv3 database (http://gong_lab.hzau.edu.cn/lncRNASNP3#!/).

### Statistical analysis bioinformatics analysis

Data from multiple independent experiments were processed using GraphPad Prism 7.0 and SPSS 20.0 software. Differences in PTCSC3 and miR-182-5p expression between the normal group and the tumor group were determined by Student’s t-test, and if more than two groups were analyzed by one-way ANOVA followed by the Tukey post hoc test. The correlation between clinical parameters of prostate cancer patients and abnormal expression of PTCSC3 was evaluated by Chi-square test. The survival status of the included prostate cancer patients was analyzed by Kaplan-Meier curve and multivariable Cox analysis, and forest plots were also created. Pearson r analysis was used to evaluate the relationship between PTCSC3 and miR-182-5p.

## Results

### PTCSC3 expression in prostate cancer and its prognostic value

**Fig. 1:**
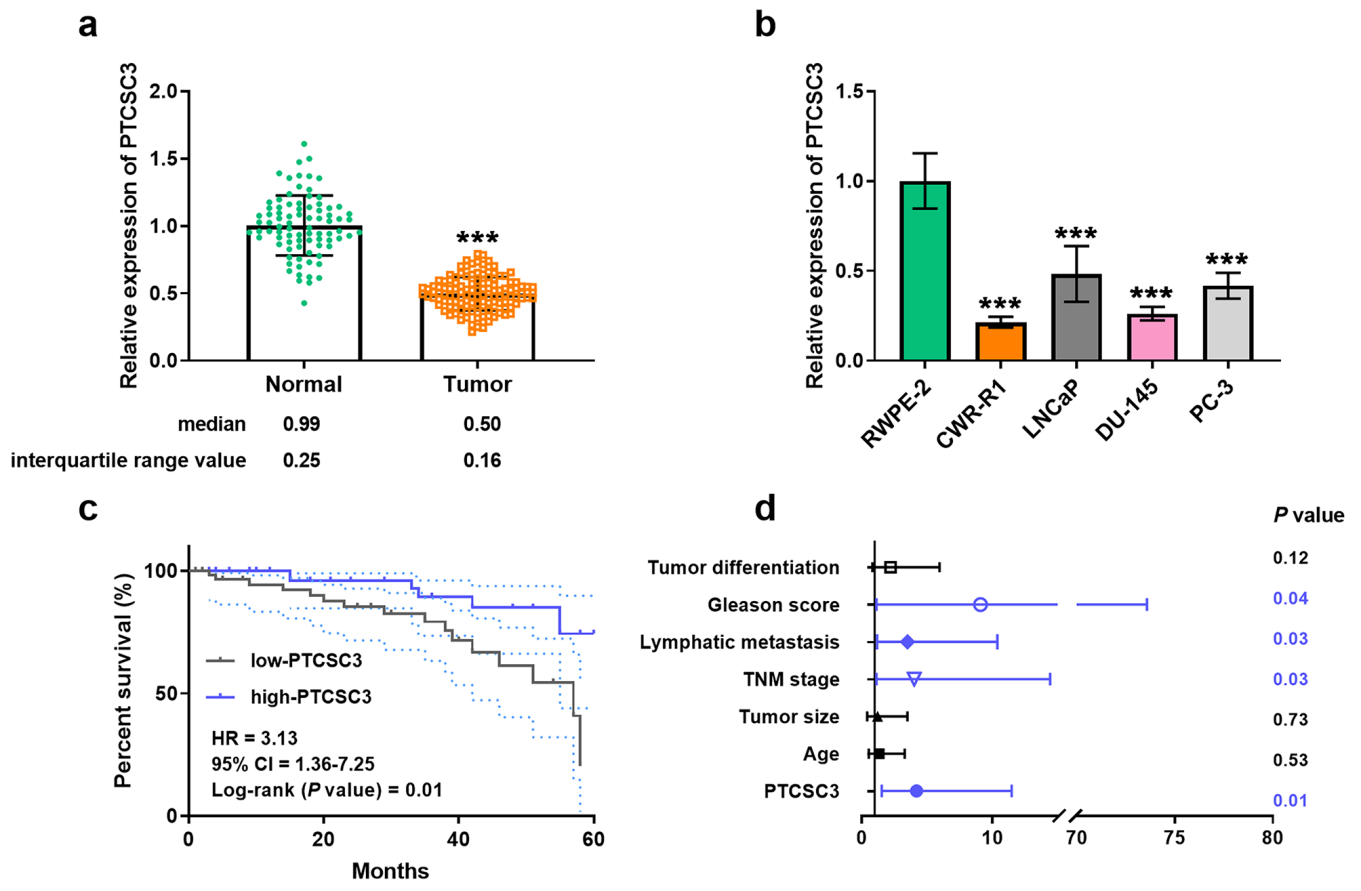
LncRNA PTCSC3 was expressed at lower levels and related to the survival outcome of patients. (**a**-**b**) PTCSC3 levels in serum and cell samples of prostate cancer by RT-qPCR assays. (**c**) The survival time of the PTCSC3 low-group was shorter than the PTCSC3 high-group via Kaplan-Meier curve (Log-rank *P* value = 0.01). (**d**) Multivariable Cox analysis confirmed that PTCSC3 was one of the independent prognostic factors of prostate cancer (*P* = 0.01). ***P < 0.001

We first assessed the levels of PTCSC3 in serum samples from 125 prostate cancer patients. In Fig. 1a, serum PTCSC3 expression in tumor patients was lower than those in the normal group. Similarly, we observed downregulation of PTCSC3 in prostate cancer cells through RT-qPCR assays (Fig. 1b). In addition, the patients were divided into low-group (*n* = 64) and high-group (*n* = 61) based on the mean expression of PTCSC3 in prostate cancer.

The collected clinical information of the patients was analyzed and recorded in Table 1 and found that low PTCSC3 level was significantly correlated with TNM stage (*P* = 0.04), lymphatic metastasis (*P* = 0.03), and Gleason score (*P* = 0.02). Prognostic data from 125 prostate cancer patients revealed that those in the PTCSC3 low-group had shorter survival times than the PTCSC3 high-group (HR = 3.13, 95% CI = 1.36–7.25, Log-rank *P* value = 0.01; Fig. 1c). Multivariable Cox analysis and related forest plots described that PTCSC3 (*P* = 0.01), TNM stage (*P* = 0.03), lymphatic metastasis (*P* = 0.03) and Gleason score (*P* = 0.04) are independent prognostic factors for prostate cancer (Fig. 1d; Table 2). These findings indicated that reduced PTCSC3 expression in prostate cancer and is closely associated with the prognosis and survival of patients.

**Table 1:** Association between PTCSC3 levels and features of prostate cancer patients

Features	Patients (n = 125)	Low group (n = 64)	High group (n = 61)	*P* value
Age (year)				0.7
≤ 60	61	30	31	
> 60	64	34	30	
Tumor size (cm)				0.5
≤ 3	86	46	40	
> 3	39	18	21	
TNM stage				0.04
I, II;	83	37	46	
III, IV	42	27	15	
Lymphatic metastasis				0.03
Negative	89	40	49	
Positive	36	24	12	
Gleason score				0.02
≤ 7	86	38	48	
> 7	39	26	13	
Tumor differentiation				0.2
Well, Moderate	84	32	38	
Poor	35	32	23	

**Table 2:** Multivariable Cox analysis of patient clinical indicators and overall survival

Items	HR	95% CI	*P*
PTCSC3	4.2	1.5–11	0.01
Age	1.3	0.6–3.3	0.5
Tumor size	1.2	0.4–3.5	0.7
TNM stage	4.0	1.1–14	0.03
Lymphatic metastasis	3.5	1.2–10	0.03
Gleason score	9.1	1.1–74	0.04
Tumor differentiation	2.3	0.8–6	0.1

### Relationship among levels of PTCSC3 and the activity and behavior of prostate cancer cells

**Fig. 2:**
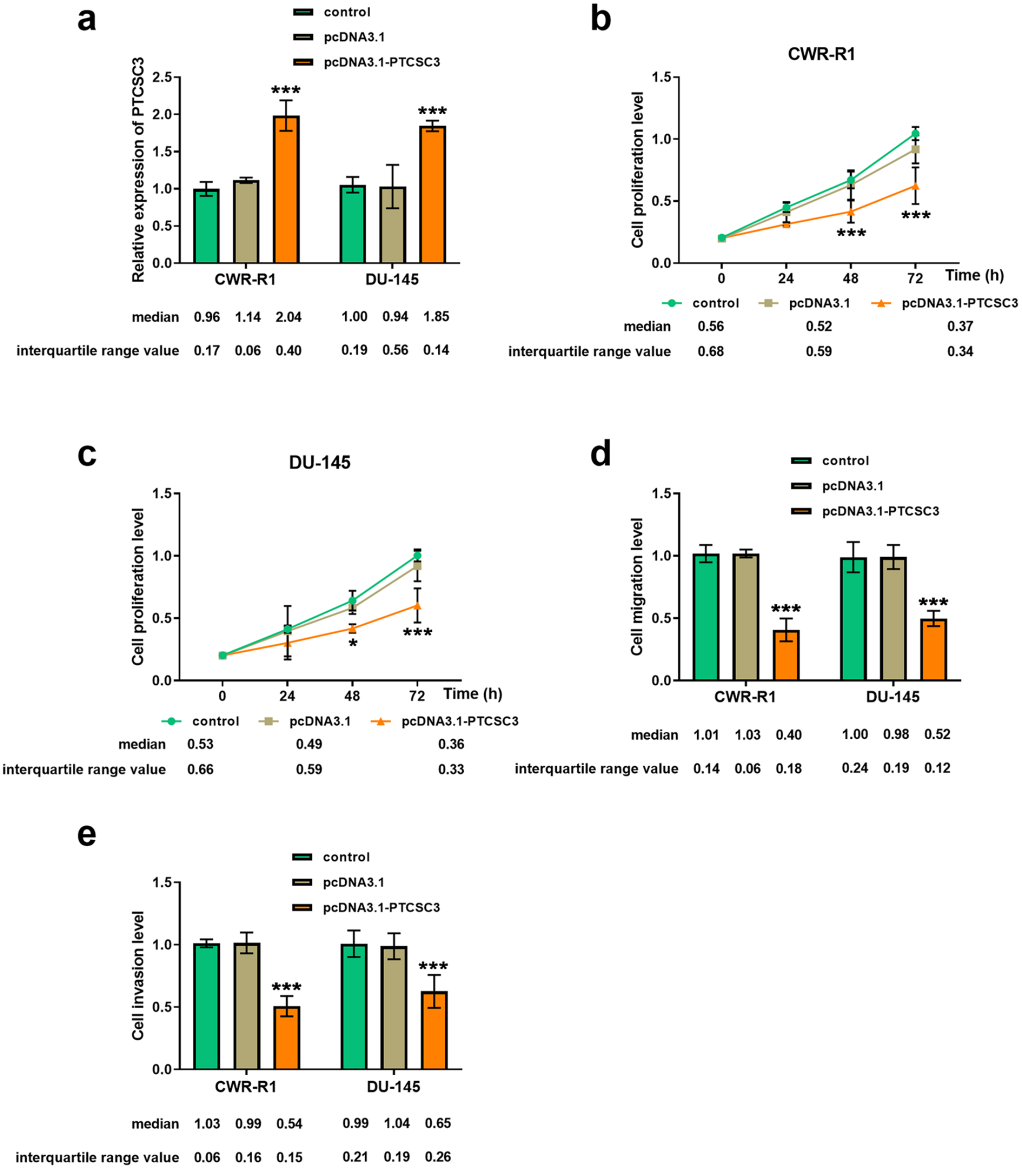
Regulation of prostate cancer cells by elevated PTCSC3. (**a**) Efficiency of transfection of pcDNA3.1-PTCSC3 in CWR-R1 and DU-145 cells. (**b**-**c**) Changes of cell growth after transfection with pcDNA3.1-PTCSC3 by CCK-8 assay. (**d**-**e**) The migratory and invasive ability of prostate cancer cells was inhibited by pcDNA3.1-PTCSC3 by Transwell assay. ***P < 0.001

We chose CWR-R1 and DU-145 prostate cancer cells with lower PTCSC3 expression for in vitro cell assays. Figure 2a demonstrates the efficiency of PTCSC3 overexpression. After transfecting with pcDNA3.1-PTCSC3, the growth activity of both CWR-R1 (Fig. 2b) and DU-145 (Fig. 2c) cells decreased, as observed in the CCK-8 assay. Transwell assay demonstrated that PTCSC3 overexpression inhibited prostate cancer cell behavior (Fig. 2d-e). It was hypothesized that high PTCSC3 levels may alleviate the progression of prostate cancer.

### Relationship between PTCSC3 and miR-182-5p expression

**Fig. 3:**
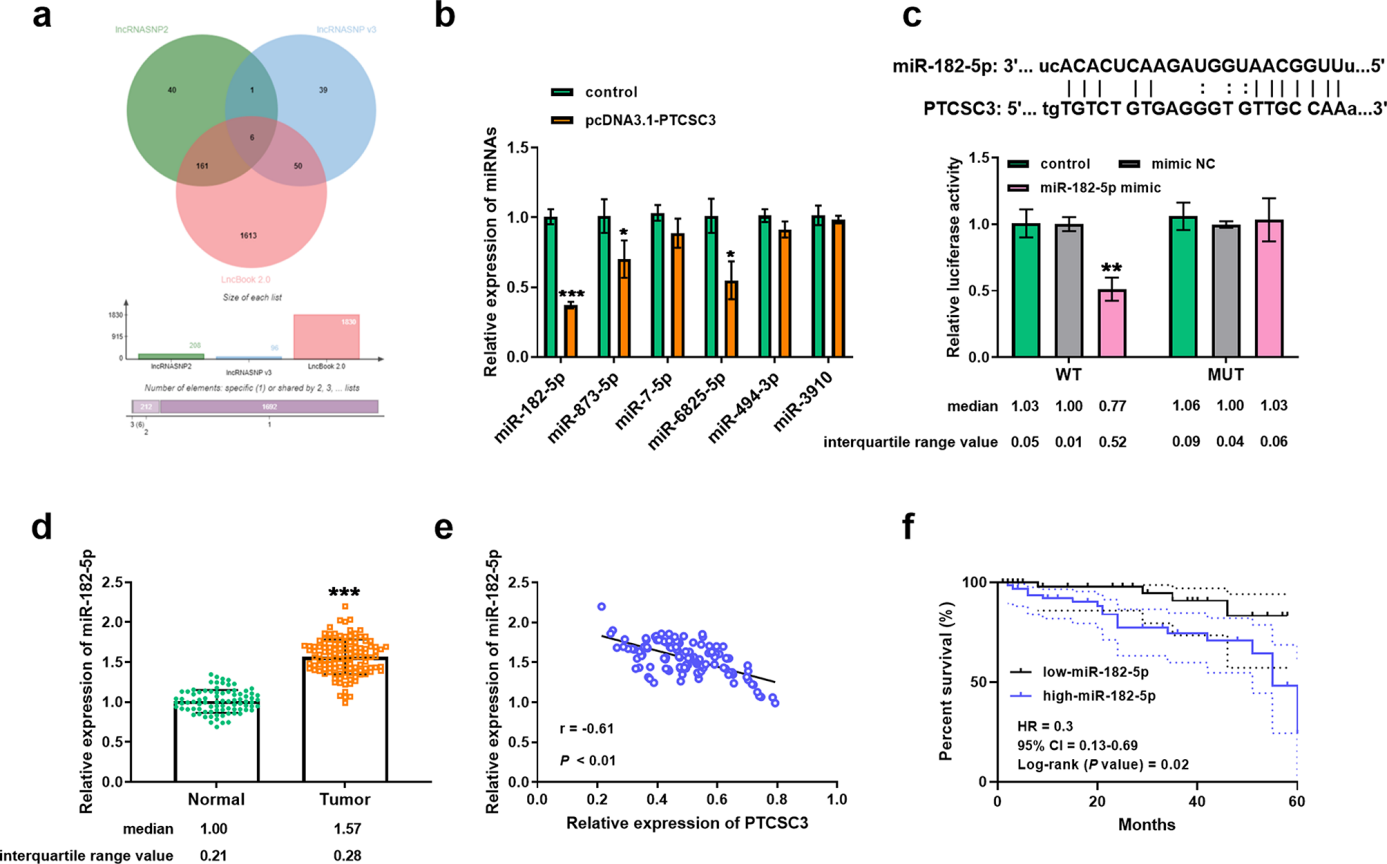
PTCSC3 targeted and negatively regulated the miR-182-5p expression. (**a**) Venn diagram for prediction of downstream target of PTCSC3 by bioinformatics. (**b**) The relative expression of different miRNAs after transfection with pcDNA3.1-PTCSC3. (**c**) Luciferase assay confirmed that PTCSC3 was sponge of miR-182-5p. (**d**) miR-182-5p levels were upregulated in prostate cancer serum samples through RT-qPCR assays. (**e**) PTCSC3 and miR-182-5p were negatively correlated by Pearson r analysis (*r* = -0.61, *P* value < 0. 01). (**f**) The survival time of the miR-182-5p low-group was longer than the miR-182-5p high-group via Kaplan-Meier curve (Log-rank *P* value = 0.02). *P < 0.05, **P < 0.01, ***P < 0.001

The downstream targets of PTCSC3 were predicted using the lncRNASNP2, lncRNASNPv3, and LncBook2.0 datasets, identifying six miRNAs (miR-182-5p, miR-873-5p, miR-7-5p, miR-6825-5p, miR-494-3p, miR-3910; Fig. 3a). RT-qPCR assays showed that the expression of miR-182-5p, miR-873-5p and miR-6825-5p were all decreased after transfection with pcDNA3.1-PTCSC3, with miR-182-5p being the most significantly downregulated (Fig. 3b). Furthermore, bioinformatics analysis revealed the connection sites between PTCSC3 and miR-182-5p. The luciferase reporter assays were conducted in CWR-R1 cells as an example, and the results showed that the miR-182-5p mimic reduced the luciferase activity of WT-PTCSC3, while the luciferase activity of MUT-PTCSC3 was not affected (Fig. 3c). Additionally, serum miR-182-5p was upregulated in 125 prostate cancer patients (Fig. 3d). A negative correlation was observed between PTCSC3 and miR-182-5p by Pearson r correlation coefficient analysis (*r* = -0.61, *P* value < 0.01; Fig. 3e). These results elaborated that PTCSC3 directly targets and downregulates miR-182-5p in prostate cancer. Kaplan-Meier curve analysis revealed a lower survival probability for patients with high-miR-182-5p expression compared to those with low-miR-182-5p expression (HR = 0.3, 95% CI = 0.13–0.69, Log-rank *P* value = 0.02; Fig. 3f).

### Co-transfection of miR-182-5p and the inhibition of pcDNA3.1-PTCSC3 on the cell

**Fig. 4:**
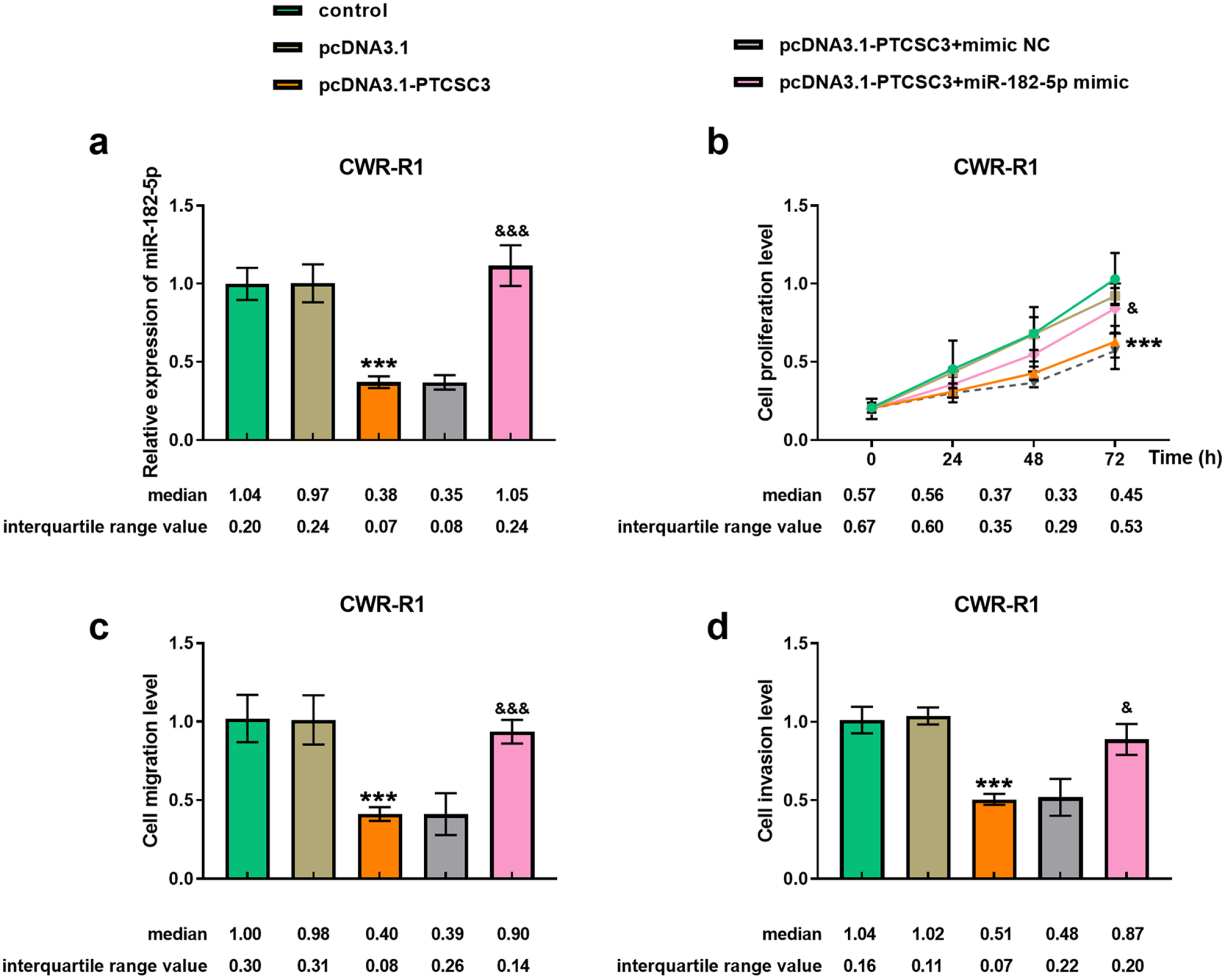
miR-182-5p mimic neutralized the inhibitory effect of pcDNA3.1-PTCSC3 on cells. (**a**) Changes in miR-182-5p level after co-transfection of pcDNA3.1-PTCSC3 and miR-182-5p mimic into CWR-R1 cells. (**b**-**d**) The involvement of high miR-182-5p level reversed the effects of pcDNA3.1-PTCSC3 on the proliferative, migratory and invasive potential of CWR-R1 cells via CCK-8 and Transwell assays. ***P < 0.001, with control; ^&^*P* < 0.05, ^&&&^*P* < 0.001, with pcDNA3.1-PTCSC3

To better understand how the PTCSC3/miR-182-5p axis regulation impacts the development of prostate cancer, CWR-R1 cells were selected for reversion assays. The changes of miR-182-5p levels after the transfection of pcDNA3.1-PTCSC3 or pcDNA3.1-PTCSC3 + miR-182-5p mimic were shown in Fig. 4a. After co-transfection of pcDNA3.1-PTCSC3 + miR-182-5p mimic into CWR-R1 cells, the inhibitory effect of PTCSC3 overexpression on cell proliferation was reversed via CCK-8 assay (Fig. 4b). Simultaneously, pcDNA3.1-PTCSC3 + miR-182-5p mimic restored the viability and mobility of CWR-R1 cells compared to cells transfected with pcDNA3.1-PTCSC3 by Transwell assay (Fig. 4c-d). These reversion assays stated the regulatory role of the PTCSC3/miR-182-5p axis in prostate cancer cells.

## Discussion

Prostate cancer is a kind of dangerous tumor with high incidence in middle-aged and elderly men, and its pathogenesis and pathological mechanism are not fully understood. Studies suggest a correlation between the onset of prostate cancer and changes in lncRNA expression [15, 16]. Consequently, exploring the pathological mechanisms of prostate cancer could reveal new therapeutic and prognostic targets.

Many lncRNAs are reported to exhibit regulatory potential in prostate cancer. For instance, Tang et al. showed that lncRNA SNHG1 is a prognostic factor closely related to the aggressive malignant behavior of prostate cancer [17]. LncRNA MIR4435‑2HG repressed the FAK/AKT/β‑catenin signaling pathway by binding ST8SIA1, thereby mediating the growth and activity of prostate cancer cells, as confirmed by Xing et al [18]. In our experiment, we quantified the PTCSC3 expression and found it to be lower in both prostate cancer serum and cell specimens compared to the control group. This finding is consistent with previous work showing PTCSC3 downregulated in thyroid cancer and triple-negative breast cancer [19, 20]. Moreover, the differential level of PTCSC3 was associated with TNM stage, lymphatic metastasis, and Gleason score by summarizing and analyzing the clinical data of the included patients. By considering the five-year survival rates and using both Kaplan-Meier curve and multivariable Cox analysis, we realized that PTCSC3 downregulation is linked to poorer long-term survival and prognosis for prostate cancer patients. Thus, PTCSC3 may be a potential prognostic biomarker for prostate cancer.

Overexpression of PTCSC3 has been found to inhibit the growth and behavior of CWR-R1 and DU-145 cells. Elevated PTCSC3 levels are also suggested to negatively influence the activity or movement of breast cancer and laryngeal squamous cell cancer cells [21, 22]. These findings suggest that PTCSC3 regulation could potentially control prostate cancer could progression. Furthermore, bioinformatics prediction and luciferase activity assays confirmed miR-182-5p was negatively regulated as a target of PTCSC3. miR-182-5p was found to be elevated in various types of tumors, including prostate cancer [23, 24], in line with our results. Meanwhile, the drawing of Kaplan-Meier curve revealed that upregulation of miR-182-5p was associated with shorter survival in prostate cancer patients. Based on the uniformity of the patient samples, there was a similarity in the survival curves obtained by PTCSC3 and miR-182-5p expression, but opposite survival trends were indicated. In the study of molecular mechanisms, when cells were co-transfected with pcDNA3.1-PTCSC3 and miR-182-5p mimic, cell proliferation ability was restored compared to cells transfected pcDNA3.1-PTCSC3. The miR-182-5p mimic also repaired the suppressive effect of pcDNA3.1-PTCSC3 on the migration and invasion of prostate cancer cells. Yao et al. proposed that miR-182-5p was the most prominently elevated miRNA in prostate cancer tissues as elucidated by RNA sequencing, and it accelerated tumor progression by mediating the ARRDC3/ITGB4 pathway [25]. In conclusion, PTCSC3 acts as a sponge for miR-182-5p in prostate cancer regulating its expression and thereby influencing tumor progression.

However, the generalizability of the results is limited due to the small sample size and the single-center nature of the study. Moreover, the absence of corresponding in vivo animal model experiments and the preliminary nature of pathological mechanism research restrict their direct application to clinical prediction.

## Conclusions

Taken together, this study elucidated that PTCSC3 was reduced in prostate cancer. PTCSC3 may mediate the progression of prostate cancer by targeting and negatively regulating miR-182-5p. When PTCSC3 was prominently expressed, it suppressed cell viability and behavior, and increased miR-182-5p levels may counteract the inhibitory effect of PTCSC3 on cells. Future studies should explore the potential of lncRNAs as therapeutic targets for prostate cancer, while enhancing the study’s credibility by including more samples and conducting animal experiments. This research underscores PTCSC3’s potential as a prognostic biomarker for prostate cancer, offering a new direction and theoretical foundation for patient treatment.

## Data Availability

The datasets used and/or analysed during the current study are available from the corresponding author on reasonable request.

## Abbreviations

lncRNAs: Long non-coding RNAs
PTCSC3: lncRNA PTCSC3
miRNAs: microRNAs
WT: Wild-type PTCSC3
MUT: Mutant-type PTCSC3
